# Endoscopic ultrasound-guided choledochoduodenostomy using single-step lumen-apposing metal stents for primary drainage of malignant distal biliary obstruction (SCORPION-p): a prospective pilot study

**DOI:** 10.1055/a-2134-3537

**Published:** 2023-08-23

**Authors:** Jeska A. Fritzsche, Paul Fockens, Marc G. Besselink, Olivier R. Busch, Freek Daams, Nahid S. M. Montazeri, Johanna W. Wilmink, Rogier P. Voermans, Roy L. J. Van Wanrooij

**Affiliations:** 1 Department of Gastroenterology and Hepatology, Amsterdam UMC, location University of Amsterdam, Amsterdam, The Netherlands; 2 Amsterdam Gastroenterology Endocrinology Metabolism, Research Institute, Amsterdam, The Netherlands; 3 Treatment and Quality of life, Cancer Center Amsterdam, Amsterdam, The Netherlands; 4 Department of Gastroenterology and Hepatology, Amsterdam UMC, location Vrije Universiteit, Amsterdam, The Netherlands; 5 Department of Surgery, Amsterdam UMC, location University of Amsterdam, Amsterdam, The Netherlands; 6 Department of Surgery, Amsterdam UMC, location Vrije Universiteit, Amsterdam, The Netherlands; 7 Department of Gastroenterology and Hepatology, Biostatistics Unit, Amsterdam UMC, location University of Amsterdam, Amsterdam, The Netherlands; 8 Department of Medical Oncology, Amsterdam UMC, location University of Amsterdam, Amsterdam, The Netherlands

## Abstract

**Background**  This study aimed to assess the safety and feasibility of endoscopic ultrasound-guided choledochoduodenostomy (EUS-CDS) using a lumen-apposing metal stent (LAMS) as a primary drainage strategy in patients with distal malignant biliary obstruction (MBO).

**Methods**  A prospective, single-center, pilot study was conducted in patients with pathology-confirmed MBO without gastric outlet obstruction. The primary outcome was technical success. Secondary outcomes included clinical success, adverse events (AEs), and reinterventions. The study was registered in the Netherlands Trial Registry (registry number NL9757).

**Results**  22 patients were enrolled (median age 69.5 years [interquartile range 64–75.3]). Technical success was achieved in 20/22 patients (91 %). AEs occurred in one patient, namely perforation following inadequate stent deployment (5 %), which was treated in the same procedure. Clinical success was achieved in 19/22 patients (86 %). Stent dysfunction was observed in 11/20 patients (55 %) after technically successful EUS-CDS: two patients were treated conservatively and nine patients underwent reintervention(s). One patient died within ≤ 30 days due to fulminant disease progression.

**Conclusions**  The results confirmed the safety and feasibility of EUS-CDS using LAMS as a primary drainage strategy. The high incidence of stent dysfunction should be improved before EUS-CDS with LAMS can be seen as a valid alternative to endoscopic retrograde cholangiopancreatography.

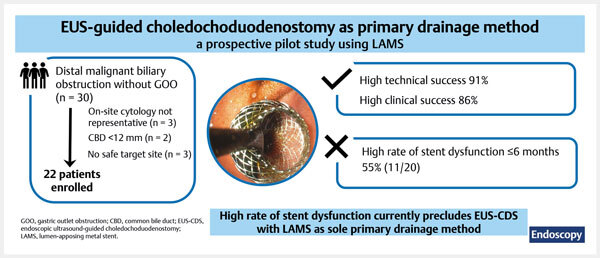

## Introduction

Endoscopic ultrasound-guided choledochoduodenostomy (EUS-CDS) is a relatively new technique that allows the endoscopist to create a biliodigestive anastomosis. As the tumor is bypassed with EUS-CDS, the effort required to obtain biliary access is more straightforward compared with endoscopic retrograde cholangiopancreatography (ERCP). Moreover, EUS-CDS obviates the need for manipulation of the papilla in order to gain biliary access, and the stent does not cause acute obstruction of the pancreatic duct, thereby precluding the risk of post-procedural pancreatitis.

 EUS-CDS has already been shown to be superior to percutaneous approaches in patients with distal malignant biliary obstruction (MBO) 1 2 3 . Based on these promising results, EUS-CDS is now also being compared with ERCP. Current prospective studies, however, all used biliary self-expandable metal stents, whereas electrocautery-enhanced lumen-apposing metal stents (LAMSs) would simplify the procedure 1 2 4 5 . 

Therefore, the aim of this prospective pilot study (SCORPION-p) was to assess the safety and feasibility of EUS-CDS using LAMS as the primary drainage strategy in patients with distal MBO.

## Methods

### Study design

Consecutive patients were screened for eligibility between October 2021 and June 2022 at Amsterdam UMC. Patients with a distal MBO confirmed by histology or cytology (including rapid onsite evaluation strongly suggestive of malignancy) and who had an indication for biliary drainage were considered eligible. The main exclusion criteria were surgically altered anatomy, cancer extending into the antrum or proximal duodenum, extensive liver metastases, World Health Organization performance score of 4, uncorrectable coagulopathy, or clinically relevant gastric outlet obstruction (GOO). The study was approved by the medical ethics committee of Amsterdam UMC. All patients provided written informed consent before inclusion. An independent monitor performed clinical trial monitoring.

### Study procedures

 All patients received a single dose of prophylactic broad-spectrum intravenous antibiotics in line with European Society of Gastrointestinal Endoscopy guideline recommendations 6 . Anticoagulants were stopped if applicable (i. e. an international normalized ratio of < 1.5 was permitted). Antiplatelet monotherapy was allowed; in cases of dual antiplatelet therapy, one of the two drugs needed to be discontinued 5 days prior to the procedure and was restarted 24 hours post-procedurally. 

**Fig. 1:**
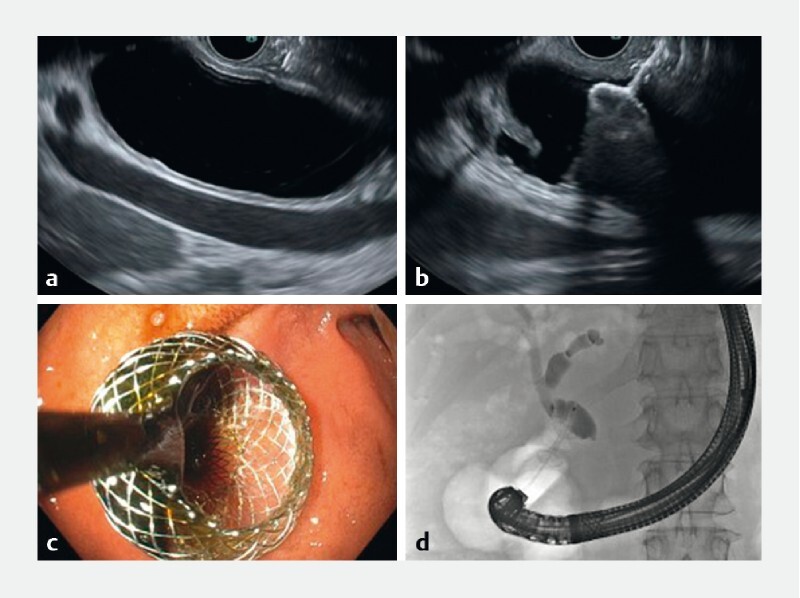
Endoscopic ultrasound-guided choledochoduodenostomy with electrocautery-enhanced lumen-apposing metal stent (LAMS) placement and confirmation of technical success by cholangiogram. **a** Sonographic identification of the common bile duct (CBD) proximal to the tumor. A target site where the CBD was ≥ 12 mm, with no intervening vessels or ascites, was identified. Using pure cutting current, the LAMS was introduced into the bile duct using the free-hand technique. **b** The distal flange was deployed in the CBD under endosonographic guidance. **c** The proximal flange was subsequently deployed under endoscopic guidance, and resulted in immediate bile flow from the LAMS.  **d** Cholangiogram via the LAMS confirmed adequate stent position.

 The procedure was performed using a linear ultrasound endoscope (Olympus GF-UCT180; Olympus Tokyo, Japan) with the patient in the left lateral or prone position. For cases without a previous tissue diagnosis, a fine-needle biopsy and/or fine-needle aspiration was performed to confirm malignant obstruction. The common bile duct (CBD) was identified proximally to the level of the tumor obstruction and at least 2 cm below the hilum. Subsequently, the origin of the cystic duct from the CBD was visualized. Care was taken to avoid intervening blood vessels. To allow safe stent deployment, the minimum bile duct diameter at the puncture site was set at 12 mm given that all procedures were performed by experts in LAMS placement 6 7 . If the diameter was < 12 mm, a standard ERCP was performed. EUS-CDS was performed using the “free-hand technique,” meaning that the electrocautery-enhanced LAMS was directly introduced into the bile duct using pure cutting current (100 W). The Hot AXIOS stent (Boston Scientific, Marlborough, Massachusetts, USA), 6 × 8 mm, was used. In cases where the LAMS catheter could not be advanced deep enough into the bile duct, a guidewire was advanced toward the hilum to redirect the catheter and facilitate further advancement. In bile ducts with a small diameter, the distal flange was deployed in a stepwise manner. The biliary system was visualized following LAMS placement by contrast injection via a diagnostic catheter through the LAMS, in order to confirm adequate stent position and exclude contrast leakage. The procedure is illustrated in Fig. 1 . Three gastroenterologists (P.F., R.P.V., R.L.J.W.), experienced in both EUS and ERCP, performed all study procedures, with two of them being present in the endoscopy suite during each procedure. 

Follow-up was performed after 2 weeks, 4 weeks, 3 months, and 6 months.

### Outcome

 The primary outcome was technical success. Secondary outcomes were: 1) clinical success, defined as at least 50 % decrease of bilirubin and/or relief of symptoms without the need for reintervention within 30 days; 2) procedure time, measured from introduction of the endoscope until visual flow of bile through the LAMS; when fine-needle biopsy or fine-needle aspiration was required, time was started after completion of this procedure; 3) (serious) adverse events (AEs) within ≤ 30 days after the procedure. Periprocedural AEs were events that occurred during the procedure. Severity of AEs was graded according to the AGREE classification 8 . Stent dysfunction was defined as recurrent jaundice (conjugated bilirubin ≥ 35 µmol/L [2.0 mg/dL]) after initial clinical success, persistent jaundice and dilatation of the bile ducts, or cholangitis. The reason for stent dysfunction was classified according to the Leuven-Amsterdam-Milan Study Group classification of EUS-CDS dysfunction 9 . Time to recurrent biliary obstruction was calculated from the moment of stent insertion until stent dysfunction. Reinterventions in cases of stent dysfunction were reported. Dysfunction-free survival was defined as the number of days after EUS-CDS until death without experiencing stent dysfunction. 

### Statistical analysis

Descriptive statistics were used to report proportions and characteristics of the results using R version 4.0.1 (R Foundation for Statistical Computing, Vienna, Austria). Categorical variables were expressed as absolute and relative frequencies, and 95 %CIs were constructed using the exact binomial distribution approximation. Continuous data were presented as medians and interquartile ranges (IQRs). (Dysfunction-free) survival was estimated using Kaplan–Meier survival analysis; as all patients who were still undergoing follow-up were censored at 6 months, only a point estimate without 95 %CI was provided.

## Results

### Baseline characteristics

 A total of 30 patients were eligible and signed the informed consent. Eight patients were excluded because rapid onsite evaluation could not confirm malignancy (n = 3), the CBD diameter was < 12 mm (n = 2), or because there was no safe target site to perform the procedure, either because the tumor was too close to the hilum (n = 2) or because of ascites (n = 1). Finally, 22 consecutive patients with distal MBO were enrolled. The full screening and selection process is depicted in **Fig. 1 s** in the online-only Supplementary material. Baseline characteristics of the included patients are summarized in Table 1 . 

**Table 1:** Baseline characteristics.

Characteristics	n = 22
Male sex, n (%)	7 (32)
Age, median (IQR), years	69.5 (64.0–75.3)
BMI, median (IQR), kg/m ^2^	24.7 (23.7–26.1)
Type of tumor, n (%)	
Pancreatic ductal adenocarcinoma	20 (91)
Duodenal carcinoma	1 (5)
Distal cholangiocarcinoma	1 (5)
WHO performance score at inclusion, n (%)	
0: Fully active	6 (27)
I: Restricted in physically strenuous activity	12 (55)
II: Ambulatory, but unable to carry out any work activities	2 (9)
III: Capable of only limited selfcare	2 (9)
Use of anticoagulant drugs, n (%)	7 (32)
Tumor stage at inclusion, n (%)	
Resectable	10 (46)
Locally advanced	6 (27)
Metastatic	6 (27)
Serum total bilirubin, median (IQR), µmol/L	225 (130.75–335.25)
Diameter of CBD on EUS, median (IQR), mm	16.5 (13.25–20.75)
Concomitant chemotherapy at inclusion, n (%)	2 (9)
Cholecystectomy prior to intervention, n (%)	4 (18)

BMI, body mass index; IQR, interquartile range; WHO, World Health Organization; CBD, common bile duct; EUS, endoscopic ultrasound.

### Technical success

Immediate technical success was achieved in 18/22 patients. In two additional patients, the distal flange was initially inadequately deployed in the bile duct wall, leading to minor bile spill, which was immediately resolved after manipulation (n = 1) or replacement with a second LAMS (n = 1), without clinical consequences. Therefore, the overall technical success rate was 91 % (20/22; 95 %CI 71 %–99 %).

In two patients the procedure was unsuccessful. In one patient, the stent was unintentionally placed in the cystic duct. In the other patient, the distal flange was deployed outside the bile duct wall. An ERCP with closure of the defect in the duodenum using a through-the-scope clip was performed in the same procedure and the patient recovered uneventfully. In one patient, a double-pigtail stent was placed through the LAMS to prevent stent obstruction by blood clots after self-limiting intraprocedural bleeding. Median procedure time was 11 minutes (IQR 7–16).

### Clinical success

Clinical success was achieved in 19/22 patients (86 %; 95 %CI 65 %–97 %). The patient in whom the stent was unintentionally placed in the cystic duct underwent a second successful EUS-CDS procedure due to inadequate biliary drainage. The other two patients underwent successful placement of an additional double-pigtail stent to achieve adequate biliary drainage because of early cholangitis (n = 1) or suspected stent obstruction (n = 1).

### Adverse events

Besides the perforation described above, which was treated endoscopically, no periprocedural AEs occurred.

 Eight patients (36 %) experienced a possible related AE ≤ 30 days after the procedure. Two AEs were unrelated to stent dysfunction: one patient had mild intermittent abdominal pain, which resolved after placement of a double-pigtail stent through the LAMS, and one patient developed rhabdomyolysis and kidney failure < 2 weeks after the procedure, which completely resolved and had unknown relation to the procedure. Six patients developed cholangitis due to stent dysfunction. None of the patients developed pancreatitis or delayed bleeding ( Table 2 ). 

**Table 2:** Adverse events ≤ 30 days including grading.

Adverse events	n = 22
Adverse events ≤ 30 days, n (%)	
Perforation	1 (5)
Pancreatitis	0 (0)
Bleeding	0 (0)
Cholangitis	6 (27)
Other	2 (9)
Intermittent abdominal pain	1 (5)
Rhabdomyolysis with kidney failure	1 (5)
Severity of adverse events ≤ 30 days, n (%) 1	
Grade II	3 (14)
Grade IIIa	5 (23)
Grade IVa	1 (5)
30-day mortality, n (%)	1 (5)

1 According to the AGREE classification [8].

 One patient died ≤ 30 days from fulminant disease progression, which was considered unlikely to be related to the procedure ( Table 2 ). 

### Stent dysfunction (n = 20)

 A total of 11/20 patients with a technically successful procedure (55 %) had experienced stent dysfunction during the 6-month follow-up, presenting with either cholangitis (n = 10) or jaundice (n = 1). Stent dysfunction occurred after a median of 6 days (IQR 5–87.5). Median estimated dysfunction-free survival was 140 days. Reason and grading of stent dysfunction is shown in **Table 1 s** . 

 In two patients, cholangitis was treated successfully with antibiotics, but in nine patients reintervention was required. Overall, endoscopic reinterventions were successful in 8/9 patients (89 %). In patients who developed GOO due to disease progression (n = 3), concomitant surgical (n = 1) or endoscopic (n = 1) gastroenterostomy was performed, or the condition was left untreated according to the patient’s wishes (n = 1). Reinterventions are summarized in **Fig. 2 s** . 

### Follow-up (n = 20)

Median total follow-up was 149 days (IQR 62.5–180). Five patients underwent surgical resection after a median of 34 days (IQR 23.5–49.75). Eight patients died after a median of 80 days (IQR 71–157). The remaining seven patients were still undergoing follow-up after 6 months. Estimated median survival was 172 days.

## Discussion

This pilot study prospectively evaluated the use of EUS-CDS with LAMS as the primary drainage strategy in patients with distal MBO. EUS-CDS showed high technical and clinical success rates in combination with minimal periprocedural AEs. The high rate of stent dysfunction (55 %), however, presents a challenge that first needs to be addressed before the potential benefits of EUS-CDS with LAMS can be realized.

 Technical and clinical success rates were comparable to previous studies performing EUS-CDS with LAMS after unsuccessful ERCP, with ranges of 89 %–100 % and 82 %–100 %, respectively 2 9 10 11 12 13 14 15 16 17 . The stent dysfunction rate in this study, however, was considerably higher compared with the 6 %–37 % reported previously for EUS-CDS with LAMS 9 10 11 12 13 14 15 16 17 . This discrepancy may be partially explained by the fact that the majority of studies on this topic were retrospective and may have underestimated the rate of stent dysfunction. Second, a relatively strict, though clinically relevant, definition of stent dysfunction was used in the current study, including cholangitis as well as persistent or recurrent jaundice. Third, despite GOO being an exclusion criterion, three patients developed GOO during the course of the disease, which may have contributed to the occurrence of cholangitis 9 18 . Fourth, the use of LAMS with a relatively small diameter (6 × 8 mm) may have contributed, as currently there is some evidence that stents with larger diameters may reduce the risk of stent dysfunction 16 . Finally, in our study, double-pigtail stents were not routinely placed through the LAMS, although recent data show that this may be beneficial 19 . On the other hand, the fact that five patients underwent surgical resection after a median of 34 days could have led to an underestimation; however, considering stent dysfunction occurred after a median of 6 days, this factor is expected to be of limited influence. Data on surgical resection after EUS-CDS are still scarce; however, we believe the available data show no reason to be reluctant to perform EUS-CDS in operable patients while awaiting further studies in this specific patient group 20 . 

 Although the rate of cholangitis due to stent dysfunction was high, the course of the disease was generally mild. The vast majority of patients were successfully treated with antibiotics and/or endoscopic reintervention. Stent dysfunction after ERCP with self-expandable metal stents, though lower than with EUS-CDS in the current study, is also substantial, with a range of 3 %–43 % 4 5 . However, with regard to other AEs, such as pancreatitis, cholecystitis, and delayed bleeding, the safety profile of EUS-CDS seems to be superior to that of ERCP 21 . Moreover, periprocedural AEs of EUS-CDS in the current study were limited, and were managed endoscopically in the same session without clinical implications. 

 EUS-CDS, using the current technique, is unable to fully replace ERCP, however, as EUS-CDS was not feasible in 17 % of our patients. In 5/30 included patients, the CBD diameter was too small (< 12 mm) or there was no safe target site at which to perform the procedure, making the patient ineligible for EUS-CDS. Lack of feasibility was mainly due to insufficient bile duct dilatation, which is in line with a recent study on pre-procedural cross-sectional imaging that identified a sufficiently (> 12 mm) dilated CBD in only 78.8 % of patients 7 . Furthermore, EUS-CDS should not be conducted in patients with GOO due to the high risk of influx of gastric contents in this specific group. Thus, endoscopists should be well trained in both EUS and ERCP in order to switch from EUS-CDS to ERCP when indicated, as well as to adequately manage periprocedural AEs. 

The findings of this study are limited by the small sample size and the lack of a control group. Future studies should directly compare the overall impact of AEs and stent dysfunction of either technique on clinical condition, quality of life, and delay or annulment of treatment. However, in order to conduct such a trial, the EUS-CDS procedure should first be further optimized to lower the risk of stent dysfunction.

In conclusion, the present study supports the safety and feasibility of EUS-CDS using LAMS as the primary drainage strategy in patients with distal MBO. However, the high incidence of stent dysfunction currently limits the use of EUS-CDS with LAMS as a valid alternative to ERCP with self-expandable metal stents. Further studies on the benefit of coaxial stent placement through the LAMS or alternative stent designs are necessary to reduce the risk of stent dysfunction.

## Table 1 s, Figs. 1 s, 2 s

Supplementary material Zusatzmaterial
