# Supplementary material for: Endoscopic ultrasound-guided choledochoduodenostomy using single-step lumen-apposing metal stents for primary drainage of malignant distal biliary obstruction (SCORPION-p): a prospective pilot study
Source: Endoscopy. 2023 Aug 23;56(1):47–52. doi: 10.1055/a-2134-3537 (PMC11321721; doi:10.1055/a-2134-3537)

Supplementary material

SUPPLEMENTARY MATERIAL

"Endoscopic ultrasound-guided choledochoduodenostomy using single-step lumen-apposing metal stents for primary drainage of malignant distal biliary obstruction (SCORPION-p): a prospective pilot study"

Jeska A. Fritzsche, Paul Fockens, Marc G. Besselink, Olivier R. Busch, Freek Daams, Nahid S.M. Montazeri, Johanna W. Wilmink, Rogier P. Voermans*, Roy L.J. Van Wanrooij*

*Contributed equally to the work

 | © 2023. Thieme. All rights reserved.

Supplementary material

Table 1s Grading and causes of stent dysfunction.

Outcome	Total (n=20)
Severity of stent dysfunction, n (%)[c]	
• Grade II	2 (10)
• Grade IIIa	8 (40)
• Grade IVa	1 (5)
Cause of stent dysfunction, n (%)[d]	
• Type 2a: Sludge impaction	1 (5)
• Type 2b: Food impaction	2 (10)
• Type 3a: LAMS compression on biliary side	4 (20)
• Type 5: GOO	3 (15)
• Unknown	1 (5)
Other interventions, n (%)	
• Percutaneous drainage of liver abscesses	1 (5)
• Diagnostic laparoscopy including percutaneous drainage of liver abscess[e]	1 (5)
• EUS-guided gastrojejunostomy	1 (5)
• Surgical gastrojejunostomy	1 (5)

IQR, interquartile range; GOO, gastric outlet obstruction; EUS, endoscopic ultrasound. [a]Missing in 1 patient who underwent resection after 7 days; [b]In 11 patients experiencing stent dysfunction; [c]According to AGREE classification [1]; [d]According to the Leuven-Amsterdam-Milan Study Group classification of EUS-CDS dysfunction [2]. [e]Liver abscess after diagnostic liver punction.

References

1. Nass KJ, Zwager LW, van der Vlugt M, Dekker E, Bossuyt PMM, Ravindran S, et al. Novel classification for adverse events in GI endoscopy: the AGREE classification. Gastrointest Endosc. 2022;95(6):1078-85.e8. doi: 10.1016/j.gie.2021.11.038.

2. Vanella G, Bronswijk M, Dell'Anna G, Voermans RP, Laleman W, Petrone MC, et al. Classification, risk factors, and management of lumen apposing metal stent dysfunction during follow-up of endoscopic ultrasound-guided choledochoduodenostomy: Multicenter evaluation from the Leuven-Amsterdam-Milan Study Group. Dig Endosc. 2023;35(3):377-88. doi: 10.1111/den.14445.

 | © 2023. Thieme. All rights reserved.

Supplementary material

Fig. 1s Screening and selection of patients for the SCORPION-p study.

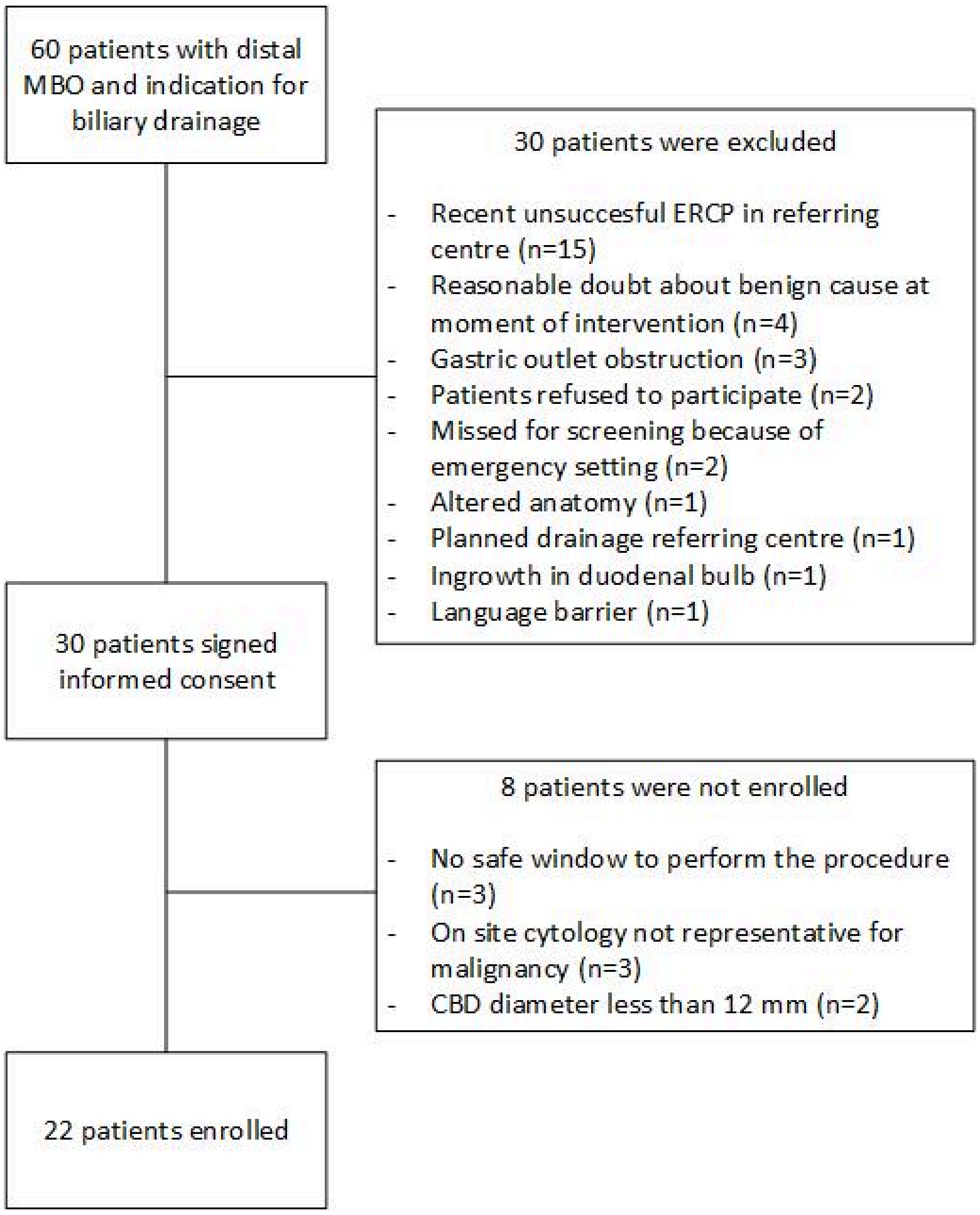

 | © 2023. Thieme. All rights reserved.

Supplementary material

Fig. 2s Reinterventions.

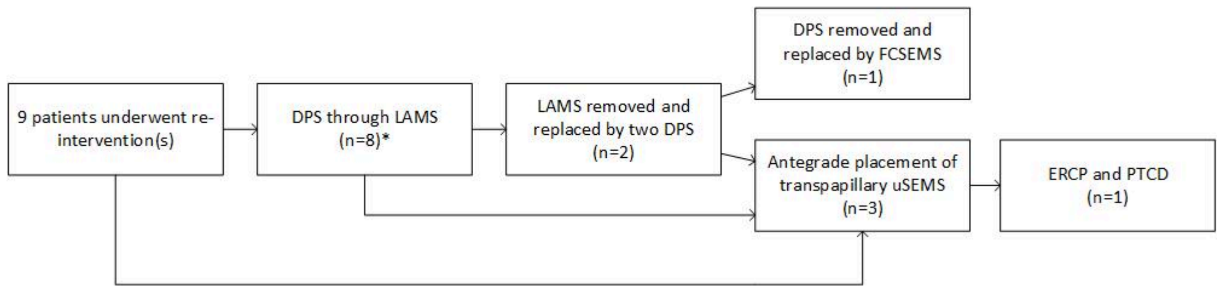

Supplement: Supplementary file 1 — Supplementary material [file 22798supmat_10-1055-a-2134-3537.pdf]
